# The evaluation of a stepped care approach for early intervention of borderline personality disorder

**DOI:** 10.1186/s40479-024-00256-1

**Published:** 2024-06-18

**Authors:** Marialuisa Cavelti, Yasmine Blaha, Stefan Lerch, Christian Hertel, Thomas Berger, Corinna Reichl, Julian Koenig, Michael Kaess

**Affiliations:** 1 University Hospital of Child and Adolescent Psychiatry and Psychotherapy, University of Bern, Bolligenstrasse 111, Bern 60, 3000 Switzerland; 2 Department of Clinical Psychology and Psychotherapy, University of Bern, Bern, Switzerland; 3 Faculty of Medicine and University Hospital Cologne, Department of Child and Adolescent Psychiatry, Psychosomatics and Psychotherapy, University of Cologne, Cologne, Germany; 4 Department of Child and Adolescent Psychiatry, University Hospital Heidelberg, Heidelberg, Germany

**Keywords:** Borderline personality disorder, Adolescence, Stepped care, Dialectical behavioral therapy, Cutting down program, Early intervention

## Abstract

**Background:**

The current study evaluated the stepped care approach applied in AtR!Sk; a specialized outpatient clinic for adolescents with BPD features that offers a brief psychotherapeutic intervention (Cutting Down Program; CDP) to all patients, followed by a more intensive Dialectical Behavioral Therapy for Adolescents (DBT-A) for those whose symptoms persist.

**Methods:**

The sample consisted of 127 patients recruited from two AtR!Sk clinics. The number of BPD criteria, psychosocial functioning, severity of overall psychopathology, number of days with non-suicidal self-injury (NSSI; past month), and the number of suicide attempts (last 3 months) were assessed at clinic entry (T0), after CDP (T1), and at 1- and 2-year follow-up (T2, T3). Based on the T1 assessment (decision criteria for DBT-A: ≥ 3 BPD criteria & ZAN-BPD ≥ 6), participants were allocated into three groups; CDP only (*n* = 74), CDP + DBT-A (eligible and accepted; *n* = 36), CDP no DBT-A (eligible, but declined; *n* = 17).

**Results:**

CDP only showed significantly fewer BPD criteria (T2: β = 3.42, *p* < 0.001; T3: β = 1.97, *p* = 0.008), higher levels of psychosocial functioning (T2: β = -1.23, *p* < 0.001; T3: β = -1.66, *p* < 0.001), and lower severity of overall psychopathology (T2: β = 1.47, *p* < 0.001; T3: β = 1.43, *p* = 0.002) over two years compared with CDP no DBT-A, while no group differences were found with regard to NSSI and suicide attempts. There were no group differences between CDP + DBT-A and CDP no DBT-A, neither at T2 nor at T3.

**Discussion:**

The findings support the decision criterion for the offer of a more intense therapy after CDP. However, there was no evidence for the efficacy of additional DBT-A, which might be explained by insufficient statistical power in the current analysis.

**Supplementary Information:**

The online version contains supplementary material available at 10.1186/s40479-024-00256-1.

## Introduction

Borderline personality disorder (BPD) in young people has become a public health priority, because it is a common, disabling, and even fatal disorder [1]. Early diagnosis and treatment (“early intervention”) for BPD is indicated to prevent or attenuate the adverse personal, social, and economic consequences. Although still controversially discussed among mental health professionals and individuals with lived experiences alike [2, 3], there is a firm evidence base suggesting that BPD is a reliable and valid diagnosis in adolescence [4]. Furthermore, evidence suggests that structured psychological therapies can result in clinically relevant improvements, particularly with regard to reduction of self-harming behavior which is a predominant feature of BPD in youth [5–8]. However, the evidence is limited by inconsistent meta-analytic findings, a low number of randomized controlled studies, high heterogeneity of samples and control interventions, and high risk of bias [9, 10].

Most adolescents with BPD pathology face multiple barriers when trying to get professional help [11], including the limited availability of evidence-based therapies that are highly specialized and lengthy, and require intensive training and clinical resources [12]. A stepped care approach to treatment delivery has been proposed to address the gap between the high demand for and the limited availability of evidence-based therapies for people with BPD [11, 13–20]. Thereby, the most effective, but least intensive treatment is delivered first, with the opportunity to step up to a more specialized and intensive treatment, based on ongoing assessment of distress, needs, and preferences. Stepped care can be applied using either a stratified or a progressive approach. The stratified approach assigns individuals to the most effective, but least intensive treatment based on an initial assessment, while the progressive approach offers the first-step treatment to all individuals, with the opportunity to step up for those who do not respond [21]. Stratified stepped care may be ultimately the most efficient approach, but it requires knowledge concerning which patient would benefit from particular interventions before the interventions are assigned. Clinical staging may inform the stepped care approach for young people with emerging BPD [5, 22–25]. It takes into account that young people often present with sub-threshold, mixed, and frequently changing symptoms that may not meet yet the diagnostic threshold, but are already associated with emotional burden, a decline in psychosocial functioning, and risk for self-harm, and determine the appropriate treatment according to the stage of the developing disorder [26]. The assumption is that early-stage treatments have a more favorable risk–benefit ratio and can be less specialized and intensive than later-stage treatments [27].

The aim of the current study was to evaluate the progressive stepped cared approach applied in AtR!Sk; a specialized outpatient service that provides evidence-based early intervention for adolescents with BPD features [7]. All patients presenting with any risk-taking or self-harming behavior receive a brief, low-intensity psychotherapeutic intervention for non-suicidal self-injury (NSSI), the Cutting Down Program (CDP) [28, 29]. The presence of any risk-taking and self-harming behavior was chosen as entry criterion into the specialized treatment program as evidence indicates that these behaviors may constitute a risk marker for BPD in adolescents [6, 7]. In line with the expert consensus that early intervention is indicated in the presence of at least 3 BPD criteria [30, 31], patients with persistent BPD symptoms (≥ 3 BPD criteria and a severity score of ≥ 6) after CDP are offered a longer, more intensive Dialectical Behavioral Therapy for Adolescents (DBT-A) [8, 32]. In the current study, we first examined the adequacy of the decision criteria for the transition from the first to the second step of treatment. This was achieved by comparing patients who were not considered eligible for DBT-A after CDP, as their BPD symptoms were below the pre-defined cut-off (i.e., “CDP only group”), with patients who were considered in need of DBT-A after CDP, as their BPD symptoms were above the pre-defined cut-off, but declined the additional therapy offer (i.e., “CDP no DBT-A group”). We hypothesized that the CDP only group would demonstrate lower levels of BPD pathology, general psychopathology, and self-harm, and higher levels of psychosocial functioning one and two years after baseline compared with the CDP no DBT-A group (hypothesis 1). Second, we examined the incremental clinical utility of the second step of treatment by comparing patients who were considered eligible for DBT-A after CDP and accepted the offer (i.e., “CDP + DBT-A group”) with the CDP no DBT-A group. We assumed that the CDP + DBT-A group would show lower levels of BPD pathology, general psychopathology, and self-harm, and higher levels of psychosocial functioning at follow-ups compared with the CDP no DBT-A group (hypothesis 2).

## Methods

### Participants and procedures

The data for the current analyses were pooled from two cohort studies. Participants were consecutively recruited from AtR!Sk (German: Ambulanz für Risikoverhalten & Selbstschädigung) at the Department of Child and Adolescent Psychiatry, University Hospital Heidelberg, Germany, and its pendant at the University Hospital of Child and Adolescent Psychiatry and Psychotherapy, Bern, Switzerland [7]. After the baseline assessment (T0) at clinic entry, all patients presenting with at least one risk-taking or self-harming behavior (i.e. NSSI, suicide attempts, alcohol or drug misuse, sexual risk behavior, delinquent behavior, truancy, and excessive media usage) irrespective of any BPD criteria receive CDP, followed by a second assessment (T1). If patients still meet three or more BPD criteria in the Structured Clinical Interview for DSM-IV-Axis II Personality Disorders (SCID-II; [33, 34]) and reach an overall severity score of 6 or higher in the Zanarini Rating Scale for BPD (ZAN-BPD; [35]), DBT-A is offered. The decision criteria were chosen in line with the expert consensus that early intervention for BPD is indicated in the presence of three or more BPD criteria [36]. Both treatments have been evaluated in randomized-controlled trials (RCTs) [28, 29, 32, 37] and are described in more detail in the Supplementary Materials (SM).

At T0, patients were invited to take part in the cohort study. Inclusion criteria were: 12–17 years of age and any type of risk-taking or self-harming behavior. Exclusion criteria were insufficient German language skills. All participants and their legal guardians (if under the age of 16 years in Germany or under the age of 14 in Switzerland, respectively) provided written informed consent (or assent, respectively) before inclusion in the study. T0 and T1 assessments were part of the routine clinical procedure. Further assessments were conducted one year (T2) and two years (T3) after baseline. All assessments were conducted by trained clinical psychologists or PhD students. Participants were reimbursed 20 Euros (AtR!Sk Heidelberg) and 85 CHF (AtR!Sk Bern), respectively, for each follow-up assessment. The studies were approved by the local ethics committees (Heidelberg: ID S-449/2013; Bern: ID 2018–00942). The cohort study in Heidelberg started in 2013 and was completed at the end of 2020. The cohort study in Bern started in November 2018 and is still running. Data release for the current study was on the 18^th^ of December 2023.

### Measures

Sociodemographic information including age and sex was assessed. The German version of the *Mini-International Neuropsychiatric Interview for Children and Adolescents* (MINI-KID; [38]) was applied to assess psychiatric diagnoses according to the DSM-IV and ICD-10. BPD features and diagnosis according to DSM-IV were measured by the German version of the SCID-II [33, 34], with well-established psychometric properties [39]. BPD diagnosis is met, if at least five of the nine BPD criteria are fulfilled. The diagnostic criteria have remained unchanged in the DSM-5. The severity of each present BPD criterion was rated with regard to the past week using the ZAN-BPD [35]*.* It is a clinician-administered scale ranging from 0 (= no symptoms) to 4 (= severe symptoms). Psychosocial functioning was assessed by the German version of the *Social and Occupational Functioning Assessment Scale* (SOFAS; [40]) in Bern, and the Global Assessment of Functioning (GAF; [41–43]) in Heidelberg. Scores range between 0 and 100, with higher scores indicating better social and educational/occupational functioning. The SOFAS differs from the GAF by focusing on psychosocial functioning independently of the severity of psychopathology. For the current analyses, a composite score “Level of Functioning (LoF)” was generated by the standardized values of the SOFAS and GAF. Z-standardization was achieved by subtracting the mean at baseline from each value, and dividing each difference by the standard deviation at baseline. The German version of the *Clinical Global Impression Scale—Severity* (CGI-S; [44]) was applied to estimate severity of overall psychopathology within the past seven days, ranging from 1 (= not ill at all) to 7 (= severely ill). The German version of the *Self-Injurious Thoughts and Behaviours Interview (SITBI-G)* [45] was used to capture the number of days with NSSI in the past month, and the number of suicide attempts in the last three months in Bern or in the past month and the last six months in Heidelberg, respectively. For participants from Heidelberg, linear interpolation was used to estimate the number of suicide attempts in the last three months, using the values for the number of suicide attempts in the past month and the last six months. The interpolated values were rounded to the next integer to assure count data. In only 3.7% of all cases differed the number of suicide attempts in the past months from the number of suicide attempts in the last six months, justifying the interpolation.

### Statistical analyses

T0 (baseline), T1 (decision criteria), and T2 (retrospective assessment of received therapy) data was necessary to test the hypotheses. Therefore, only participants who completed all three assessments were included in the analyses. There were five participants, who were below the cut-off for DBT-A after CDP, but did nonetheless receive DBT-A due to clinical considerations. Those five participants were allocated to the CDP only group for testing hypothesis 1, and to the CDP + DBT-A group for testing hypothesis 2. Backward stepwise logistic regression minimizing the Bayesian Information Criterion (BIC) was conducted to explore differences between participants who were and were not lost to follow-up with regard to age, sex (female, male), place of recruitment (Heidelberg, Bern), number of BPD criteria (SCID-II), psychosocial functioning (LoF), severity of overall psychopathology (CGI-S), number of suicide attempts (last three months; SITBI-G), and number of days with NSSI (past month; SITBI-G).

To test hypothesis 1, separate mixed-effect linear regressions for the number of BPD criteria (SCID-II), psychosocial functioning (LoF), and severity of overall psychopathology (CGI-S) were conducted, with time (T0, T1, T2, T3), group (CDP only, CDP + DBT-A, CDP no DBT-A), the interaction time x group, and the control variables age, sex (female, male), place of recruitment (Heidelberg, Bern), and other therapy than DBT-A (yes / no) between T1 and T2, and between T2 and T3, respectively, as fixed effects, and subject ID as random effect. For the number of suicide attempts (last three months; SITBI-G) and the number of days with NSSI (past month; SITBI-G), mixed-effect Poisson regression and mixed-effect negative binomial regression, respectively, were applied, using the same fixed and random effects as described above. The decision for either Poisson or negative binomial regression for count data was based on a model comparison using the Akaike Information Criterion (AIC) and BIC, with lower values indicating better model fit (not reported). Before analysis, one outlier with unrealistic values for the number of suicide attempts (i.e., 8, 0, 30, and 30 suicide attempts within the last three months at T0, T1, T2, and T3), and one patient with an impossible value for the number of days with NSSI (i.e., 40 days with NSSI within the past month) were excluded. For all outcome variables, contrasts between the CDP only group and the CDP + no DBT-A group at T1 and T2 were conducted.

Two additional post-hoc analyses were conducted to get a deeper insight into the findings: Firstly, to better understand who benefitted from the CDP in the first step — what might have had an impact on whether patients received the DBT-A offer and whether they accepted or declined it -, the differential trajectories between T0 and T1 were explored across groups. This was achieved by contrasts comparing each outcome variable at T1 versus T0 for each group separately. Secondly, to assess the suitability of the decision criteria used in the AtR!Sk progressive stepped care approach for a future stratified stepped care model, we calculated the proportion of patients allocated to either the CDP only or the two DBT-A groups (i.e., CDP + DBT-A, CDP no DBT-A) who had already met the criteria at baseline.

To test hypotheses 2, the mixed-effect linear regressions for the number of BPD criteria (SCID-II), psychosocial functioning (LoF), and severity of overall psychopathology (CGI-S) described above were repeated, including the outcome variable at T0 and T1 as additional fixed effect. For the number of suicide attempts (last three months; SITBI-G) and the number of days with NSSI (path month; SITBI-G), Poisson and negative binomial regressions, respectively, were conducted, based again on model comparisons using AIC and BIC (not reported). In the prediction model for the number of suicide attempts the random effect was omitted, as it was estimated to be zero. Contrasts between the CDP + DBT-A group and the CDP no DBT-A group at T1 and T2 were calculated.

All statistical analyses were conducted using Stata 17 software [46]. The significance level was set at α = 0.05.

## Results

### Participants

Of *N* = 925 participants (Heidelberg: *n* = 673, Bern: *n* = 252) assessed at T0, *n* = 208 (Heidelberg: *n* = 79, Bern: *n* = 129) took part in the T1 assessment, *n* = 500 (Heidelberg: *n* = 351, Bern: *n* = 149) in the T2 assessment, and *n* = 373 (Heidelberg: *n* = 263, Bern: *n* = 110) in the T3 assessment. The analysis of the losses to follow-up revealed that those who did not attend the T1 assessment (*n* = 717) were more likely to have ICD-10 F8 (i.e., intellectual disabilities) diagnoses (OR = 15.23, *p* = 0.009, 95% CI = 1.96, 117.94) and less likely to be assessed in Bern (OR = 0.13, *p* < 0.001, 95% CI = 0.09, 0.19) compared to those who did attend (*n* = 208). No differences were found between those who missed the T2 assessment (*n* = 425) and those who completed it (*n* = 500). Finally, participants who did not complete the T3 assessment (*n* = 552) were more like to have ICD-10 F9 (i.e., behavioral and emotional disorders with onset usually occurring in childhood and adolescence, such as attention deficit hyperactivity disorder (ADHD) and conduct disorder) diagnoses (OR = 1.63, *p* = 0.003, 95% CI = 1.18, 2.27) compared to those who did (*n* = 373).

There were *n* = 127 participants (Heidelberg: *n* = 43, Bern: *n* = 84) who took part in the T0, T1, and T2 assessments, and were, thus, included in the current analyses. Thereof, *n* = 74 participants (Heidelberg: *n* = 23, Bern: *n* = 51) completed the T3 assessment. The analysis of the losses to follow-up revealed that those who did not attend the T3 assessment (*n* = 53) were more likely to have ICD-10 F6 diagnoses (i.e., disorders of adult personality and behavior; OR = 3.2, *p* = 0.004, 95% CI = 1.45, 7.04) compared with those who did (*n* = 74). Of the 127 participants, 79 were assigned to the CDP only group (74 for testing hypothesis 2), 31 to the CDP + DBT-A group (36 for testing hypothesis 2), and 17 to the CDP + no DBT group. Sample characteristics at baseline for the three groups and for the total sample are provided in Table 1. Mean and standard deviations of the outcome variables (i.e., number of BPD criteria, psychosocial functioning (LoF), severity of overall psychopathology (CGI-S), number of suicide attempts (last 3 months), number of days with NSSI (past month) per group and time point (T0, T1, T2, T3) are depicted in Table 1 in the SM. Notably, at baseline, 19 patients did not meet any BPD criteria, 23 patients did not report NSSI in the past month, and 5 patients did neither meet any BPD criteria nor report NSSI in the past month and were, therefore, offered CDP for other risk-taking behaviors.

**Table 1:** Sample characteristics at baseline

	CDP only (*n* = 74)	CDP + DBT-A (*n* = 36)	CDP + no DBT-A (*n* = 17)	Total (*N* = 127)
Age, M (SD)	15.04 (1.63)	15.53 (1.61)	15.24 (1.39)	15.20 (1.60)
Sex, n (%)				
Female	70 (94.6)	34 (94.4)	16 (94.1)	120 (94.5)
Male	4 (5.4)	2 (5.6)	1 (5.9)	7 (5.5)
Place of recruitment, n (%)				
Heidelberg (D)	23 (31.1)	10 (27.8)	10 (58.8)	43 (33.9)
Bern (CH)	51 (68.9)	26 (72.2)	7 (41.2)	84 (66.1)
Number of BPD criteria, M (SD)	2.04 (1.80)	4.97 (2.36)	4.47 (1.74)	3.20 (2.39)
Days with NSSI (past month)				
M (SD)	6.08 (7.67)	5.39 (5.50)	7.71 (9.51)	6.10 (7.38)
Md (IQR)	3.00 (7.00)	3.50 (7.00)	4.00 (11.00)	3.00 (7.00)
Number of suicide attempts (last 3 months)				
M (SD)	0.16 (0.52)	0.50 (1.38)	0.29 (0.59)	0.28 (0.87)
Md (IQR)	0.00 (0.00)	0.00 (1.00)	0.00 (0.00)	0.00 (0.00)
Psychosocial functioning (LoF)^a^, M (SD)	0.30 (0.94) / *n* = 72	-0.23 (0.93) / *n* = 35	-0.21 (0.79)	0.08 (0.95) / *N* = 124
Severity of overall psychopathology (CGI-S), M (SD)	4.11 (0.93) / *n* = 72	4.74 (0.95) / *n* = 35	5.00 (0.61)	4.41 (0.96) / *N* = 124
ICD-10 diagnoses, n (%)^2^				
F0	0	0	0	0
F1	7 (9.5)	9 (25.0)	3 (17.6)	19 (15.0)
F2	0	0	0	0
F3	48 (64.9)	28 (77.8)	15 (88.2)	91 (71.7)
F4	33 (44.6)	21 (58.3)	7 (41.2)	61 (48.0)
F5	2 (2.7)	3 (8.3)	1 (5.9)	6 (4.7)
F6	11 (14.9)	22 (61.1)	6 (35.3)	39 (30.7)
F7	0	0	0	0
F8	0	1 (2.8)	0	1 (0.8)
F9	11 (14.9)	5 (13.9)	7 (41.2)	23 (18.1)
Other therapy than DBT-A between T1 and T2, n (%)	30 (40.5)	0 (0)	9 (52.9)	39 (30.7)
Other therapy than DBT-A between T2 and T3, n (%)	21 (46.7) / *n* = 45	0 (0)	4 (44.4) / *n* = 9	25 (27.8) / *N* = 90

The 5 participants who received DBT-A even though they did not meet the criteria are allocated to the CDP + DBT-A group

*BPD* borderline personality disorder, *CDP* Cutting Down Program, *CGI-S* Clinical Global Impression Scale – Severity, *DBT-A* Dialectical Behavioral Therapy for Adolescents, *IQR* interquartile range, *LoF* Level of Functioning, *M* mean, *Md* median, *NSSI* non-suicidal self-injury, *T1* after CDP, *T2* 1-year follow-up, *T2* 2-year follow-up, *SD* standard deviation, *F0* Organic, including symptomatic, mental disorders, *F1* Mental and behavioral disorders due to psychoactive substance use, *F2* Schizophrenia, schizotypal, delusional, and other non-mood psychotic disorders, *F3* Affective disorders, *F4* Neurotic, stress-related and somatoform disorders, *F5* Behavioral syndromes associated with physiological disturbances and physical factors, *F6* Disorders of adult personality and behavior, *F7* Intellectual disabilities, *F8* Pervasive and specific developmental disorders, *F9* Behavioral and emotional disorders with onset usually occurring in childhood and adolescence

^a^The reported values of psychosocial functioning are z-standardized scores of the SOFAS and the GAF, respectively. To enhance interpretability, the means, SD and respective values of z = -1, z = 0 and z = 1 are reported here: SOFAS: M = 62.38, SD = 12.18, SOFAS_z = -1_ = 50.21, SOFAS_z = 0_ = 62.39, SOFAS_z = 1_ = 74.57; GAF: M = 49.49, SD = 11.33, GAF_z = -1_ = 38.15, GAF_z = 0_ = 49.49, GAF_z = 1_ = 60.81

### Hypothesis 1: testing the decision criteria for DBT-A as second step treatment

**Fig. 1:**
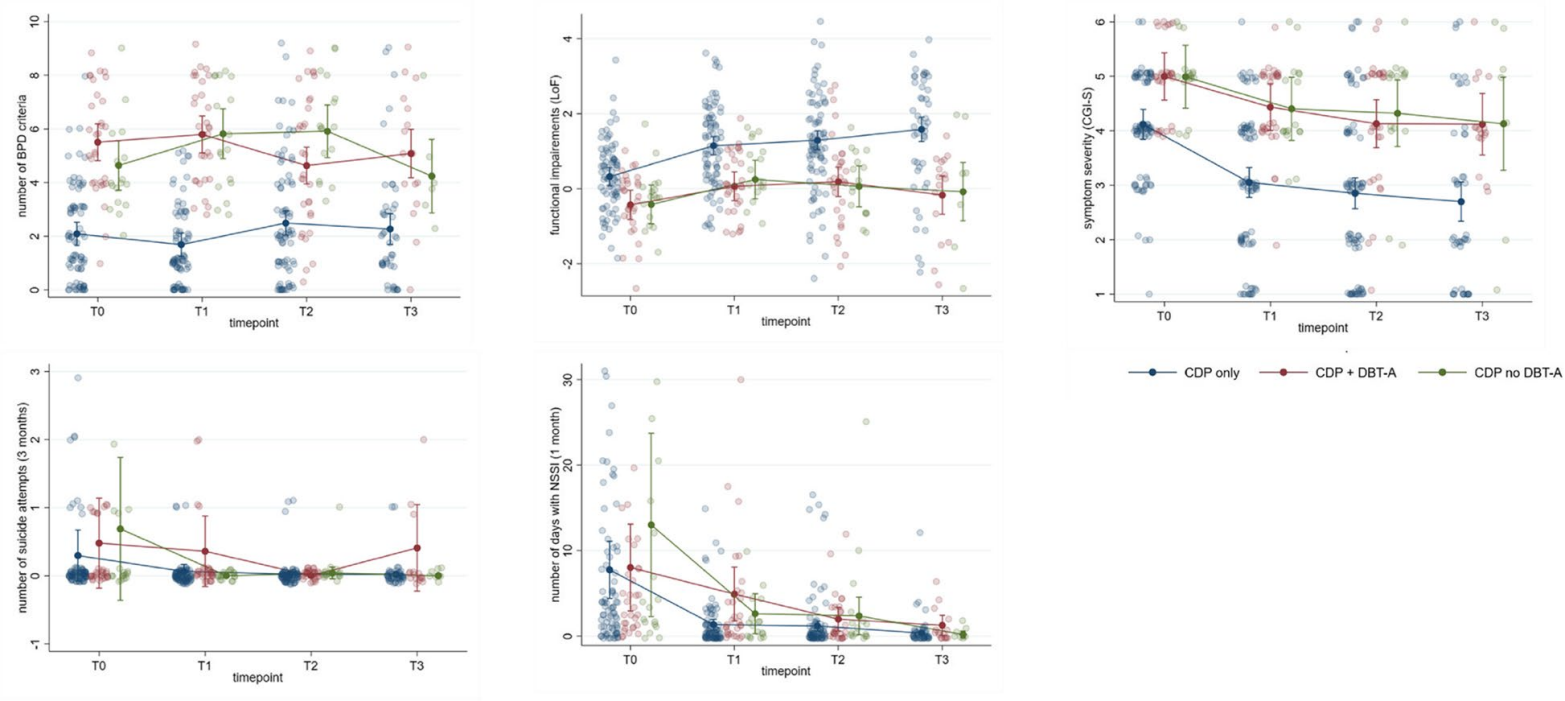
Courses of outcome variables over time (T0 = baseline, T1 = after CDP, T2 = 1-year follow-up, T3 = 2-year follow-up) separated by group (CDP only, CDP + DBT-A, CDP no DBT-A). CDP = Cutting Down Program; DBT-A Dialectical Behavioral Therapy for Adolescents; BPD = borderline personality disorder; LoF = Level of Functioning; CGI-S = Clinical Global Impression Scale – Severity; NSSI = non-suicidal self-injury. Shaded points represent the raw data point with additional 5% spherical jitter. The connected solid points represent the marginal predicted mean by the model with the 95% confidence interval

Figure 1 shows the trajectories of the three groups over time separately for each outcome variable, and Table 2 provides full results from the regression analyses testing whether the CDP no DBT-A group demonstrates higher levels of psychopathology and lower levels of psychosocial functioning one and two years after baseline compared with the CDP only group. The effects of control variables are reported in Table 2 of the SM. As depicted in Table 2, the regression models for all outcome variables were statistically significant, with the exception of the model predicting the number of suicide attempts in the last three months (with and without inclusion of the outlier). Contrasts revealed that the CDP only group had significantly fewer BPD criteria, higher levels of psychosocial functioning, and lower severity of overall psychopathology at both T2 and T3 compared with the CDP no DBT-A group, while no group differences were found with regard to the number of days with NSSI in the past month. To ensure that the five patients who showed neither NSSI nor BPD criteria at baseline did not affect the results, we repeated the analyses excluding these five patients. The findings regarding the group differences (i.e., contrasts between the CDP only group and the CDP + no DBT-A group) did not change.

**Table 2:** Results from regression analyses testing whether the CDP no DBT-A group demonstrates higher levels of psychopathology and lower levels of psychosocial functioning one and two years after baseline compared with the CDP only group (hypothesis 1)

Outcome	Model fit	Number of observations	Main effects		Contrasts: CDP only versus CDP no DBT-A				
						β	SE	p	95% CI
Number of BPD criteria	χ^2^(15) = 212.87, *p* < 0.001	446	Time	χ^2^ (3) = 4.52, *p* = 0.210	T2	3.42	0.53	< 0.001	2.37, 4.47
			Group	χ^2^ (2) = 134.52, *p* < 0.001	T3	1.97	0.74	0.008	0.51, 3.42
			Time x group	χ^2^ (6) = 26.31, *p* < 0.001					
Psychosocial functioning (LoF)	χ^2^(15) = 145.96, *p* < 0.001	439	Time	χ^2^ (3) = 29.37, *p* < 0.001	T2	-1.23	0.30	< 0.001	-1.81, -0.64
			Group	χ^2^ (2) = 65.23, *p* < 0.001	T3	-1.66	0.42	< 0.001	-2.49, 0.84
			Time x group	χ^2^ (6) = 11.17, *p* = 0.083					
Severity of overall psychopathology (CGI-S)	χ^2^(15) = 164.20, *p* < 0.001	439	Time	χ^2^ (3) = 44.05, *p* < 0.001	T2	1.47	0.33	< 0.001	0.81, 2.12
			Group	χ^2^ (2) = 56.62, *p* < 0.001	T3	1.43	0.46	0.002	0.52, 2.34
			Time x group	χ^2^ (6) = 5.49, *p* = 0.483					
Number of suicide attempts (last 3 months)	χ^2^(15) = 23.75, *p* = 0.069	440	Time	-	T2	-	-	-	-
			Group	-	T3	-	-	-	-
			Time x group	-					
Number of days with NSSI (past month)	χ^2^(15) = 141.38, *p* < 0.001	440	Time	χ^2^ (3) = 73.06, *p* < 0.001	T2	0.69	0.51	0.172	-0.30, 1.68
			Group	χ^2^ (2) = 7.14, *p* = 0.028	T3	-0.66	1.23	0.592	-3.07, 1.75
			Time x group	χ^2^ (6) = 11.86, *p* = 0.065					

*BPD* borderline personality disorder, *CDP* Cutting Down Program, *CGI- S* Clinical Global Impression Scale – Severity, *DBT-A* Dialectical Behavioral Therapy for Adolescents, *LoF* Level of Functioning

The first post-hoc analysis exploring the differential trajectories of the groups between T0 and T1 revealed that the CDP only group showed significant improvements in psychosocial functioning (*p* < 0.001), severity of overall psychopathology (*p* < 0.001), and NSSI (*p* < 0.001), with no significant change in the number of BPD criteria. The CDP no DBT-A group showed significant improvements in psychosocial functioning (*p* = 0.036), and NSSI (*p* = 0.001), with no significant change in the severity of overall psychopathology, and a significant increase in the number of BPD criteria (*p* = 0.031). In contrast, the CDP + DBT-A group showed significant improvements in psychosocial functioning (*p* = 0.038), and severity of overall psychopathology (*p* = 0.027), with no changes in the number of BPD criteria and NSSI. As the main model for suicide attempts (including the interaction time x group) was not significant, we aimed to examine the main effects only by rerunning the analysis without the interaction time x group. This simplified model revealed a significant improvement in suicide attempts between T0 and T1 for all groups (*p* = 0.004). Full results are given in Table 3 of the SM.

For the second post-hoc analysis only data of 100 patients could be used, as 27 patients had missing values in the ZAN-BPD at baseline. Of the 71 patients assigned to the CDP only group at T1, 58 (82%) did not reach the cut-off for DBT-A at baseline, while 13 patients (18%) did. In addition, of the 29 patients assigned to one of the DBT-A groups at T1, 25 (86%) met the cut-off for DBT-A at baseline, while 4 patients (14%) did not.

### Hypothesis 2: testing the benefit of DBT-A as second step treatment

Table 3 shows full results from the regression analyses testing whether CDP + DBT-A group shows lower levels of psychopathology and higher levels of psychosocial functioning one and two years after baseline compared with the CDP no DBT-A group. All models were statistically significant, with the exception of the model predicting the number of suicide attempts in the last three months. Contrasts revealed no significant group differences at T1 or T2. The effects of control variables are reported in Table 4 of the SM.

**Table 3:** Results from regression analyses testing whether CDP + DBT-A group shows lower levels of psychopathology and higher levels of psychosocial functioning one and two years after baseline compared with the CDP no DBT-A group (hypothesis 2)

Outcome	Model fit	Number of observations	Contrasts: CDP + DBT-A versus CDP no DBT-A				
				β	SE	p	95% CI
Number of BPD criteria	χ^2^(11) = 88.70, *p* < 0.001	192	T2	-1.10	0.68	0.104	-2.43, 0.23
			T3	0.94	0.88	0.285	-0.79, 2.67
Psychosocial functioning (LoF)	χ^2^(11) = 70.12, *p* < 0.001	181	T2	0.13	0.39	0.733	-0.64, 0.90
			T3	0.20	0.53	0.706	-0.83, 1.23
Severity of overall psychopathology (CGI-S)	χ^2^(11) = 66.23, *p* < 0.001	181	T2	-0.13	0.42	0.757	-0.94, 0.69
			T3	-0.36	0.52	0.492	-1.38, 0.66
Number of suicide attempts (last 3 months)	χ^2^(11) = 18.57, *p* = 0.069	188	T2	-	-	-	-
			T3	-	-	-	-
Number of days with NSSI (past month)	χ^2^(11) = 33.64, *p* < 0.001	188	T2	0.09	0.73	0.905	-1.12, 1.61
			T3	1.89	1.41	0.182	-0.89, 4.66

*BPD* borderline personality disorder, *CDP* Cutting Down Program, *CGI- S* Clinical Global Impression Scale – Severity, *DBT-A* Dialectical Behavioral Therapy for Adolescents, *LoF* Level of Functioning

## Discussion

This study examined the progressive stepped care approach applied in AtR!Sk; a specialized outpatient service for adolescents with risk-taking and self-harming behavior that offers CDP, followed by DBT-A for those with persistent symptoms. Two main findings emerged.

First, adolescents who were not considered in need of more additional treatment after brief CDP showed fewer BPD symptoms, higher levels of psychosocial functioning, and lower severity of overall psychopathology over two years compared with those who declined DBT-A after CDP even though they were considered eligible due to elevated BPD symptoms. No group differences were found in NSSI or suicide attempts at follow-ups. We interpret this finding in support of the decision criterion for DBT-A as second-step treatment that was chosen in line with the expert consensus that early intervention for BPD is indicated in the presence of three or more BPD criteria [36]. This interpretation is further supported by the post-hoc analyses, which demonstrated that both the CDP only and CDP no DBT-A groups experienced a reduction of self-harming behavior in the period of CDP delivery, while this was not the case for the CDP + DBT-A group. Notably, there was no change in BPD symptoms in the CDP only and CDP + DBT-A groups and even a worsening of these symptoms in the CDP no DBT-A group in the period of CDP delivery. Taken together, it appears that for adolescents that primarily show self-harming behavior the brief and low intense CDP specifically addressing NSSI seems to be sufficient for many individuals. This may be partially explained by impaired inhibitory control, which correlates with NSSI and contributes to the superiority of shorter treatments over longer ones [47, 48]. In contrast, adolescents that demonstrate more severe difficulties in self and interpersonal functioning (as indicated by higher mean levels of BPD symptoms in the CDP no DBT-A group and the CDP + DBT-A group) are in need of a more comprehensive therapy addressing the core of BPD [49]. This is in also in line with findings of a community-based study [50], which identified two distinct pathways to self-harm; a “psychopathological pathway” with emotion dysregulation, bullying, and caregivers’ emotional challenges from an early age, and an “adolescent risky behavior pathway” with risky behavior and less security with peers/family emerging with adolescence. It could be assumed that CDP is enough for patients on the risky behavior pathway, but not for those on the psychopathological pathway. Finally, it could be hypothesized that the lack of improvement in BPD symptoms during the period of CDP delivery in the CDP no DBT-A group may have contributed to their rejection of the DBT-A offer because they were no longer confident that therapy could help them. This interpretation is in line with evidence suggesting that reducing hopelessness and fostering the belief that change is possible is an important factor for long-term improvement among patients with BPD features [51, 52]. Further research is warranted to investigate whether the chosen decision criteria could be used for a stratified stepped care approach, differentiating those adolescents requiring CDP for self-harming behavior from those requiring a more intense and comprehensive treatment such as DBT-A for emerging BPD directly after clinic entry. Preliminary evidence for the suitability of the chosen decision criteria for a stratified stepped care approach was given by the post-hoc analysis suggesting that the majority of patients would have been allocated to the same group if the criteria had been applied at clinic entry (T0) instead of after the completion of the first step treatment (at T1).

Second, no clinical differences were found at the follow-ups between adolescents who accepted and those who declined DBT-A after CDP. This finding is somewhat surprising as both groups showed a clear indication for early intervention for BPD and DBT-A has been found to be effective in reducing BPD features in adolescents in the short-term [32, 37, 53–55]. Approximately half of the adolescents in the CDP no DBT-A group received treatment outside AtR!Sk, which may have contributed to the non-significant group differences in clinical outcomes at the follow-ups. Another explanation is the lack of power in the current study due to the small sizes of the groups to whom DBT-A was offered, suggesting that the finding has to be interpreted with caution. However, with enough power, the effect may be detectable, but still small, calling into question the cost/benefit yield of DBT-A as the step-up intervention, as it requires intensive training and clinical resources. As both groups (i.e., adolescents who accepted and those who declined DBT-A) exhibited still clinically relevant BPD features and psychosocial impairments two years after baseline (see Table 1 in the SM), further research is warranted to examine the incremental efficacy of more intense care and what kind of therapy could be a scalable and effective option for the step-up offer. Finally, the finding highlights the need to identify (early) non-responders [56], as the CDP + DBT-A group did not seem to benefit from either the CDP or the DBT-A.

### Limitations and direction for future research

The current study examined a stepped-care approach in the treatment of adolescents with BPD features in a naturalistic design. As a consequence, group allocation occurred not at random, which may have contributed to group differences in clinical variables. To adjust for this, we considered subject matching based on the propensity score, but discarded this approach because of insufficient overlap between groups. Future studies using innovative, experimental designs to examine adaptive interventions (e.g., Sequential, Multiple Assignment, Randomized Trial Design [57]) in the context of early intervention for BPD are warranted. The analyses of the losses to follow-up suggest that adolescents with ICD-10 F8 (i.e., intellectual disabilities) and F9 diagnoses (e.g., ADHD or conduct disorders) were underrepresented in the sample, restricting generalizability of findings. A major limitation of the study is the small size of the CDP + DBT-A and the CDP no DBT-A groups, limiting statistical power to detect group differences. Future studies with larger samples are required to corroborate our findings and to investigate the utility of the three BPD criteria as cut-off for a stratified stepped care approach that assigns individuals to the most effective, but least intensive treatment immediately after the assessment at clinic entry. Furthermore, future studies should consider additional or alternative decision criteria beyond the number of BPD symptoms. A primary candidate could be the degree of self- and interpersonal dysfunction or psychosocial deficits, as the main goal of early intervention for BPD involves prevention of serious health, social, and educational/occupational impairment [58]. Another important factor is age, given that early intervention is effective across adolescence, but manifests differently, necessitating more developmentally adapted therapeutic interventions [59].

## Conclusions

The current study provides evidence for three BPD criteria as cut-off for specialized and more intense early intervention comprehensively addressing difficulties in self and interpersonal functioning, while for those presenting primarily with self-harming behavior a short-term problem-focused intervention may be sufficient. While the current findings support CDP as an efficient and scalable option for the first-step treatment, no evidence was found for the efficacy of DBT-A as the step-up treatment.

### Supplementary Information

Supplementary Material 1.

## Data Availability

The data that support the findings of this study are available on request from the corresponding author.

## References

[1] Chanen AM, Sharp C, Hoffman P, Global Alliance for Prevention and Early Intervention for Borderline Personality Disorder (2017). Prevention and early intervention for borderline personality disorder: a novel public health priority. World Psychiatry..

[2] Cavelti M, Sharp C, Chanen AM, Kaess M. Commentary: commentary on the Twitter comments evoked by the May 2022 debate on diagnosing personality disorders in adolescents. Child Adolesc Ment Health. 2023;28:86–191. 10.1111/camh.12618 36478638

[3] Elvins R, Kaess M (2022). Editorial: should child and adolescent mental health professionals be diagnosing personality disorder in adolescence?. Child Adoles Ment Health.

[4] Sharp C (2017). Bridging the gap: the assessment and treatment of adolescent personality disorder in routine clinical care. Arch Dis Child.

[5] Chanen AM, Nicol K, Betts JK, Thompson KN (2020). Diagnosis and treatment of borderline personality disorder in young people. Curr Psychiatry Rep.

[6] Blaha Y, Cavelti M, Lerch S, Steinhoff A, Koenig J, Kaess M. Risk-taking and self-harm behaviors as markers of adolescent borderline personality disorder. Eur Child Adolesc Psychiatry. 2024. 10.1007/s00787-023-02353-y. [cited 2024 May 13]. 10.1007/s00787-023-02353-y PMC11272750 38194081

[7] Kaess M, Ghinea D, Fischer-Waldschmidt G, Resch F (2017). Die Ambulanz für Risikoverhalten und Selbstschädigung (AtR!Sk) – ein Pionierkonzept der ambulanten Früherkennung und Frühintervention von Borderline-Persönlichkeitsstörungen. Prax Kinderpsychol Kinderpsychiatr.

[8] Kothgassner OD, Goreis A, Robinson K, Huscsava MM, Schmahl C, Plener PL (2021). Efficacy of dialectical behavior therapy for adolescent self-harm and suicidal ideation: a systematic review and meta-analysis. Psychol Med.

[9] Wong J, Bahji A, Khalid-Khan S (2020). Psychotherapies for adolescents with subclinical and borderline personality disorder: a systematic review and meta-analysis. Can J Psychiatry.

[10] Jørgensen MS, Storebø OJ, Stoffers-Winterling JM, Faltinsen E, Todorovac A, Simonsen E (2021). Psychological therapies for adolescents with borderline personality disorder (BPD) or BPD features—a systematic review of randomized clinical trials with meta-analysis and Trial Sequential Analysis. PLoS ONE.

[11] Choi-Kain LW, Albert EB, Gunderson JG (2016). Evidence-based treatments for borderline personality disorder: implementation, integration, and stepped care. Harv Rev Psychiatry.

[12] Wall K, Kerr S, Sharp C (2021). Barriers to care for adolescents with borderline personality disorder. Curr Opin Psychol.

[13] Grenyer BFS (2014). An integrative relational step-down model of care: the project air strategy for personality disorders. ACPARIAN.

[14] Grenyer BFS, Lewis KL, Fanaian M, Kotze B (2018). Treatment of personality disorder using a whole of service stepped care approach: a cluster randomized controlled trial. van Wouwe JP, editor. PLoS ONE..

[15] Huxley E, Lewis KL, Coates AD, Borg WM, Miller CE, Townsend ML (2019). Evaluation of a brief intervention within a stepped care whole of service model for personality disorder. BMC Psychiatry.

[16] Kramer U, Kolly S, Charbon P, Ilagan GS, Choi-Kain LW (2022). Brief psychiatric treatment for borderline personality disorder as a first step of care: adapting general psychiatric management to a 10-session intervention. Personal Disord.

[17] Laporte L, Paris J, Bergevin T, Fraser R, Cardin J-F (2018). Clinical outcomes of a stepped care program for borderline personality disorder: clinical outcomes of a stepped care program for borderline personality disorder. Personal Ment Health.

[18] McGowan NM, Syam N, McKenna D, Pearce S, Saunders KEA (2021). A service evaluation of short-term mentalisation based treatment for personality disorder. BJPsych open.

[19] Paris J (2013). Stepped care: an alternative to routine extended treatment for patients with borderline personality disorder. PS.

[20] Pigot M, Miller CE, Brockman R, Grenyer BFS (2019). Barriers and facilitators to the implementation of a stepped care intervention for personality disorder in mental health services. Personal Ment Health.

[21] Nicholas J, Ringland KE, Graham AK, Knapp AA, Lattie EG, Kwasny MJ (2019). Stepping up: predictors of ‘Stepping’ within an iCBT stepped-care intervention for depression. IJERPH.

[22] Chanen AM, Nicol K (2021). Five failures and five challenges for prevention and early intervention for personality disorder. Curr Opin Psychol.

[23] Hutsebaut J, Videler AC, Verheul R, Van Alphen SPJ (2019). Managing borderline personality disorder from a life course perspective: Clinical staging and health management. Personal Disord Theory Res Treat.

[24] Hutsebaut J, Debbané M, Sharp C (2020). Designing a range of mentalizing interventions for young people using a clinical staging approach to borderline pathology. Borderline Personal Disord Emot Dysregul..

[25] Seiffert N, Cavelti M, Kaess M (2020). Klinische Stadienmodelle in der Früherkennung und -behandlung der Borderline-Persönlichkeitsstörung. Psychotherapeut.

[26] Cross S, Hickie I. Transdiagnostic stepped care in mental health. Public Health Res Pr. 2017;27. Available from: http://www.phrp.com.au/?post_type=article&p=36589. 2022 Dec 1]. 10.17061/phrp2721712 28474049

[27] Scott J, Leboyer M, Hickie I, Berk M, Kapczinski F, Frank E (2013). Clinical staging in psychiatry: a cross-cutting model of diagnosis with heuristic and practical value. Br J Psychiatry.

[28] Kaess M, Edinger A, Fischer-Waldschmidt G, Parzer P, Brunner R, Resch F (2020). Effectiveness of a brief psychotherapeutic intervention compared with treatment as usual for adolescent nonsuicidal self-injury: a single-centre, randomised controlled trial. Eur Child Adolesc Psychiatry.

[29] Rockstroh F, Edinger A, Josi J, Fischer-Waldschmidt G, Brunner R, Resch F (2023). Brief psychotherapeutic intervention compared with treatment as usual for adolescents with nonsuicidal self-injury: outcomes over a 2–4-year follow-up. Psychother Psychosom..

[30] Chanen AM, Thompson KN (2018). Early intervention for personality disorder. Curr Opin Psychol.

[31] Kaess M, Fischer-Waldschmidt G, Resch F, Koenig J (2017). Health related quality of life and psychopathological distress in risk taking and self-harming adolescents with full-syndrome, subthreshold and without borderline personality disorder: rethinking the clinical cut-off?. Borderline Personal Disord Emot Dysregul..

[32] Buerger A, Fischer-Waldschmidt G, Hammerle F, von Auer K, Parzer P, Kaess M (2019). Differential change of borderline personality disorder traits during dialectical behavior therapy for adolescents. J Pers Disord.

[33] First M, Spitzer R, Gibbon M, Williams J, Benjamin L (1994). Structured Clinical Interview for DSM-IV Axis II personality disorders (SCID-II).

[34] Fydrich T, Renneberg B, Schmitz B, Wittchen H-U (1997). Strukturiertes Klinisches Interview für DSM-IV-Achse II: Persönlichkeitsstörungen [Structured Clinical Interview for DSM-IV - Axis II: Personality Disorders].

[35] Zanarini MC (2003). Zanarini Rating Scale For Borderline Personality Disorder (ZAN-BPD): a continuous measure of DSM-IV borderline psychopathology. J Pers Disord.

[36] Chanen AM, McCutcheon LK, Germano D, Nistico H, Jackson HJ, McGorry PD (2009). The HYPE Clinic: an early intervention service for borderline personality disorder. J Psychiatr Pract.

[37] Mehlum L, Tørmoen AJ, Ramberg M, Haga E, Diep LM, Laberg S (2014). Dialectical behavior therapy for adolescents with repeated suicidal and self-harming behavior: a randomized trial. J Am Acad Child Adolesc Psychiatry.

[38] Sheehan DV, Sheehan KH, Shytle RD, Janavs J, Bannon Y, Rogers JE (2010). Reliability and validity of the mini international neuropsychiatric interview for children and adolescents (MINI-KID). J Clin Psychiatry.

[39] Carcone D, Tokarz VL, Ruocco AC (2015). A systematic review on the reliability and validity of semistructured diagnostic interviews for borderline personality disorder. Canadian Psychology / Psychologie canadienne.

[40] Goldman HH, Skodol AE, Lave TR (1992). Revising axis V for DSM-IV: a review of measures of social functioning. Am J Psychiatry.

[41] American Psychiatric Association (1994). Diagnostic and Statistical Manual of Mental Disorders, Fourth Edition (DSM-IV).

[42] Goldman HH, Skodol AE, Lave TR (1992). Revising axis V for DSM-IV: a review of measures of social functioning. AJP.

[43] Vatnaland T, Vatnaland J, Friis S, Opjordsmoen S (2007). Are GAF scores reliable in routine clinical use?. Acta Psychiatr Scand.

[44] Guy W (1976). ECDEU Assessment Manual for Psychopharmacology.

[45] Fischer G, Ameis N, Parzer P, Plener PL, Groschwitz R, Vonderlin E (2014). The German version of the self-injurious thoughts and behaviors interview (SITBI-G): a tool to assess non-suicidal self-injury and suicidal behavior disorder. BMC Psychiatry.

[46] StataCorp (2021). Stata Statistical Software: Release 17.

[47] Traynor JM, McMain S, Chapman AL, Kuo J, Labrish C, Ruocco AC (2024). Pretreatment cognitive performance is associated with differential self-harm outcomes in 6 v. 12-months of dialectical behavior therapy for borderline personality disorder. Psychol Med..

[48] Allen KJD, Hooley JM (2015). Inhibitory control in people who self-injure: Evidence for impairment and enhancement. Psychiatry Res.

[49] Gunderson JG, Herpertz SC, Skodol AE, Torgersen S, Zanarini MC (2018). Borderline personality disorder. Nat Rev Dis Primers.

[50] Uh S, Dalmaijer ES, Siugzdaite R, Ford TJ, Astle DE (2021). Two pathways to self-harm in adolescence. J Am Acad Child Adolesc Psychiatry.

[51] Mehlum L, Ramleth R-K, Tørmoen AJ, Haga E, Diep LM, Stanley BH (2019). Long term effectiveness of dialectical behavior therapy versus enhanced usual care for adolescents with self-harming and suicidal behavior. J Child Psychol Psychiatry.

[52] Mehlum L (2021). Mechanisms of change in dialectical behaviour therapy for people with borderline personality disorder. Curr Opin Psychol.

[53] Jørgensen MS, Storebø OJ, Stoffers-Winterling JM, Faltinsen E, Todorovac A, Simonsen E (2021). Psychological therapies for adolescents with borderline personality disorder (BPD) or BPD features—a systematic review of randomized clinical trials with meta-analysis and Trial Sequential Analysis. Kaess M, editor. PLoS ONE..

[54] Mehlum L, Ramleth R, Tørmoen AJ, Haga E, Diep LM, Stanley BH (2019). Long term effectiveness of dialectical behavior therapy versus enhanced usual care for adolescents with self-harming and suicidal behavior. J Child Psychol Psychiatr.

[55] Wong J, Bahji A, Khalid-Khan S. Psychotherapies for adolescents with subclinical and borderline personality disorder: a systematic review and meta-analysis. Can J Psychiatry. 2020;65(1):5–15. 10.1177/0706743719878975 PMC6966252 31558033

[56] Reichl C, Rockstroh F, Lerch S, Fischer-Waldschmidt G, Koenig J, Kaess M (2023). Frequency and predictors of individual treatment outcomes (response, remission, exacerbation, and relapse) in clinical adolescents with nonsuicidal self-injury. Psychol Med.

[57] Kidwell KM, Almirall D (2023). Sequential, multiple assignment randomized trial designs. JAMA.

[58] Hutsebaut J, Clarke SL, Chanen A. The diagnosis that should speak its name: why it is ethically right to diagnose and treat personality disorder during adolescence. Front Psychiatry. 2023;14. Available from: https://www.frontiersin.org/journals/psychiatry/articles/10.3389/fpsyt.2023.1130417/full. [cited 2024 Mar 12]. 10.3389/fpsyt.2023.1130417 PMC10203159 37229381

[59] Kaess M, Thomson M, Lerch S, Koenig J, Fischer-Waldschmidt G, Reichl C, et al. Age dependent effects of early intervention in borderline personality disorder in adolescents. Psychol Med. 2024;12:1–9. 10.1017/S0033291724000126 PMC11413336 38343374

