# Supplementary material for: The evaluation of a stepped care approach for early intervention of borderline personality disorder
Source: Borderline Personal Disord Emot Dysregul. 2024 Jun 18;11:12. doi: 10.1186/s40479-024-00256-1 (PMC11184763; doi:10.1186/s40479-024-00256-1)
Supplement: Supplementary file 1 — Supplementary Material 1. [file 40479_2024_256_MOESM1_ESM.docx]

**Supplementary Materials for**

**“The evaluation of a stepped care approach for early intervention of borderline personality disorder”**

*Interventions*

The CDP is a manualized, 10-session cognitive-behavioral intervention that has been specifically developed to reduce NSSI in adolescents. It is one of the few evidence-based interventions for this patient group ^1^. In a RCT comparing CDP with treatment as usual (TAU), both treatments were equally effective in reducing the frequency of NSSI, but the treatment effect occurred faster in the CDP group compared with the TAU group, even though the former group received fewer therapy sessions than the latter ^2^. In the AtR!Sk clinic, the CDP program is also applied to patients who do not report NSSI, but risk-taking behavior, such as alcohol or drug misuse, sexual risk behavior, delinquent behavior, truancy, and excessive media usage.

DBT-A includes 25 individual and 20 group therapy sessions and has been found to be effective in reducing self-harm and BPD symptoms pre-post, with mixed findings regarding the maintenance of the effects and functional improvements ^3–6^.

During both CDP and DBT-A, patients received family therapy sessions, psychiatric management, and specialist crisis involvement (e.g., outpatient crisis interventions or time-limited admission to the acute ward) when necessary.

***Table 1****.* Mean and standard deviation for the outcome variables per group and time point

|  | **T0** | | |  | **T1** | | |  | **T2** | | |  | **T3** | | | |  |
| --- | --- | --- | --- | --- | --- | --- | --- | --- | --- | --- | --- | --- | --- | --- | --- | --- | --- |
|  | CDP only (n = 74) | CDP + DBT-A (n = 36) | CDP + No DBT (n = 17) |  | CDP  only (n = 74) | CDP + DBT-A (n = 36) | CDP + No DBT (n = 17) |  | CDP  only (n = 74) | CDP + DBT-A (n = 36) | CDP + No DBT (n = 17) |  | CDP  only (n = 45) | CDP + DBT-A (n = 20) | CDP + No DBT (n = 9) |  |  |
| Number of BPD criteria, M (SD) | 2.04 (1.80) | 4.97 (2.36) | 4.47 (1.74) |  | 1.65 (1.47) | 5.14 (2.46) | 5.65 (1.97) |  | 2.40 (2.02) | 4.78 (2.67) | 6.06 (1.84) |  | 2.02 (2.01) | 5.10 (2.65) | 4.29 (1.89) |  |  |
| Days with NSSI (past month) , M (SD) | 6.08 (7.67) | 5.39 (5.50) | 7.71 (9.51) |  | 1.84 (5.29) | 3.86 (6.10) | 1.88 (2.83) |  | 1.49 (3.40) | 2.41 (4.23) | 2.94 (6.51) |  | 0.46 (1.92) | 1.30 (1.98) | 0.14 (0.38) |  |  |
| Number of suicide attempts (last 3 months) , M (SD) | 0.16 (0.52) | 0.50 (1.38) | 0.29 (0.59) |  | 0.04 (0.20) | 0.17 (0.51) | 0.00 (0.00) |  | 0.04 (0.20) | 0.88 (5.14) | 0.06 (0.25) |  | 0.05 (0.22) | 1.70 (6.68) | 0.00 (0.00) |  |  |
| Psychosocial functioning (LoF)^1^, M (SD) | 0.30 (0.94) | -0.23 (0.93) | -0.21 (0.79) |  | 1.19 (1.10) | 0.14 (0.82) | 0.45 (0.77) |  | 1.25 (1.27) | 0.25 (1.30) | 0.09 (0.86) |  | 1.58 (1.50) | 0.00 (1.41) | -0.03 (1.64) |  |  |
| Severity of overall psychopathology (CGI-S) , M (SD) | 4.11 (0.93) | 4.74 (0.95) | 5.00 (0.61) |  | 3.00 (1.34) | 4.28 (0.91) | 4.41 (0.87) |  | 2.83 (1.47) | 4.09 (1.36) | 4.44 (1.09) |  | 2.59 (1.53) | 4.10 (1.12) | 4.14 (1.95) |  |  |
| ***Note.*** Mean and SD of primary outcomes at each time of assessment (T0 = baseline, T1 = after CDP, T2 = 1-year follow-up, T3 = 2-year follow-up) separated by group (CDP only, CDP + DBT-A, CDP no DBT-A). CDP = Cutting Down Program; DBT-A Dialectical Behavioural Therapy for Adolescents; BPD = borderline personality disorder; LoF = Level of Functioning; CGI-S = Clinical Global Impression Scale – Severity; NSSI = non-suicidal self-injury. ^1^The reported values are z-standardized scores of the SOFAS and the GAF, respectively. To enhance interpretability, the means, SD, and respective values of z = -1, z = 0 and z = 1 are reported here: SOFAS: M = 62.38, SD = 12.18, SOFAS_z = -1_ = 50.21, SOFAS_z = 0_ = 62.39, SOFAS_z = 1_ = 74.57; GAF: M = 49.49, SD = 11.33, GAF_z = -1_ = 38.15, GAF_z = 0_ = 49.49, GAF_z = 1_ = 60.81. | | | | | | | | | | | | | | | | | |

***Table 2****.* Effect of control variables of all models conducted in hypothesis 1.

| **Outcome** | **Model fit** | **Number of observations** | **Control variables** | | | | |
| --- | --- | --- | --- | --- | --- | --- | --- |
|  |  |  |  | β | SE | p | 95% CI |
| Number of BPD criteria | χ^2^(15) = 212.87, p < 0.001 | 446 | Age | 0.17 | 0.08 | *0.037* | 0.01, 0.33 |
|  |  |  | Sex (male) | -1.14 | 0.54 | *0.036* | -2.20, -0.07 |
|  |  |  | Dataset (Bern) | 0.41 | 0.27 | 0.130 | -0.12, 0.95 |
|  |  |  | Therapy (Yes) | 0.48 | 0.30 | 0.114 | -0.11, 1.07 |
| Psychosocial functioning (LoF) | χ^2^(15) = 145.96, p < 0.001 | 439 | Age | 0.02 | 0.04 | 0.611 | -0.06, 0.11 |
|  |  |  | Sex | 0.18 | 0.29 | 0.544 | -0.39, 0.75 |
|  |  |  | Dataset (Bern) | -0.67 | 0.15 | *<0.001* | -0.96, -0.38 |
|  |  |  | Therapy (Yes) | -0.25 | .17 | 0.148 | -0.59, 0.09 |
| Severity of overall psychopathology (CGI-S) | χ^2^(15) = 164.20, p < 0.001 | 439 | Age | -0.00 | 0.05 | 0.970 | -0.10, 0.10 |
|  |  |  | Sex | -0.45 | 0.34 | 0.183 | -1.12, 0.21 |
|  |  |  | Dataset (Bern) | -0.13 | 0.17 | 0.434 | -0.47, 0.20 |
|  |  |  | Therapy (Yes) | 0.16 | 0.19 | 0.401 | -0.21, 0.53 |
| Number of suicide attempts (last 3 months) | χ^2^(15) = 23.75, p = 0.069 | 440 | Age | - | - | - | - |
|  |  |  | Sex | - | - | - | - |
|  |  |  | Dataset (Bern) | - | - | - | - |
|  |  |  | Therapy (Yes) | - | - | - | - |
| Number of days with NSSI (past month) | χ^2^(15) = 141.38, p < 0.001 | 440 | Age | 0.00 | 0.07 | 0.972 | -0.14, 0.15 |
|  |  |  | Sex | -1.32 | 0.55 | *0.017* | -2.41, -0.23 |
|  |  |  | Dataset (Bern) | 0.50 | 0.25 | *0.046* | 0.01, 0.10 |
|  |  |  | Therapy (Yes) | 0.42 | 0.34 | 0.219 | -0.25, 1.08 |
| ***Note.*** BPD = borderline personality disorder; LoF = Level of Functioning; CGI-S = Clinical Global Impression Scale – Severity; NSSI = non-suicidal self-injury. | | | | | | | |

***Table 3.*** Results of the post-hoc contrasts exploring the differential trajectories of the groups between T0 and T1

| **Outcome** | **Contrasts: T1 vs. T0** | | | | |
| --- | --- | --- | --- | --- | --- |
|  | Group | β | SE | p | 95% CI |
| Number of BPD criteria | CDP only | -0.41 | 0.25 | 0.110 | -0.90, 0.09 |
|  | CDP + DBT-A | 0.29 | 0.40 | 0.473 | -0.50, 1.08 |
|  | CDP no DBT-A | 1.18 | 0.55 | 0.031 | 0.11, 2.25 |
| Psychosocial functioning (LoF) | CDP only | 0.82 | 0.15 | <0.001 | 0.53, 1.12 |
|  | CDP + DBT-A | 0.49 | 0.24 | 0.038 | 0.03, 0.96 |
|  | CDP no DBT-A | 0.66 | 0.32 | 0.036 | 0.04, 1.28 |
| Severity of overall psychopathology (CGI-S) | CDP only | -1.07 | 0.16 | <0.001 | -1.38, -0.75 |
|  | CDP + DBT-A | -0.56 | 0.25 | 0.027 | -1.06, -0.06 |
|  | CDP no DBT-A | -0.59 | 0.34 | 0.084 | -1.26, 0.08 |
| Number of suicide attempts (last 3 months) | CDP only | -1.10 | 0.38 | 0.004 | -1.85, -0.34 |
|  | CDP + DBT-A | -1.10 | 0.38 | 0.004 | -1.85, -0.34 |
|  | CDP no DBT-A | -1.10 | 0.38 | 0.004 | -1.85, -0.34 |
| Number of days with NSSI (past month) | CDP only | -1.76 | 0.25 | <0.001 | -2.25, -1.27 |
|  | CDP + DBT-A | -0.49 | 0.36 | 0.172 | -1.19, 0.21 |
|  | CDP no DBT-A | -1.61 | 0.50 | 0.001 | -2.58, -0.63 |

***Table 4****.* Effect of control variables of all models conducted in hypothesis 2.

| **Outcome** | **Model fit** | **Number of observations** | **Control variables** | | | | |
| --- | --- | --- | --- | --- | --- | --- | --- |
|  |  |  |  | β | SE | p | 95% CI |
| Number of BPD criteria | χ^2^(11) = 88.70, p < 0.001 | 192 | Age | -0.01 | 0.12 | 0.906 | -0.24, 0.21 |
|  |  |  | Sex (male) | -0.77 | 0.79 | 0.329 | -2.32, 0.78 |
|  |  |  | Dataset (Bern) | 0.61 | 0.39 | 0.117 | -0.15, 1.37 |
|  |  |  | Therapy (yes) | 0.63 | 0.35 | 0.071 | -0.05, 1.31 |
|  |  |  | Number of BPD criteria T0 | 0.16 | 0.13 | 0.350 | -0.13, 0.37 |
|  |  |  | Number of BPD criteria T1 | 0.12 | 1.67 | 0.390 | -1.84, 4.72 |
| Psychosocial functioning (LoF) | χ^2^(11) = 70.12, p < 0.001 | 181 | Age | -0.012 | 0.06 | 0.846 | -0.14, 0.11 |
|  |  |  | Sex | 0.24 | 0.44 | 0.588 | -0.62, 1.10 |
|  |  |  | Dataset (Bern) | -0.18 | 0.24 | 0.443 | -0.65, 0.28 |
|  |  |  | Therapy (yes) | -0.33 | 0.22 | 0.130 | -0.77, 0.10 |
|  |  |  | LoF T0 | 0.31 | 0.12 | *0.008* | 0.08, 0.54 |
|  |  |  | LoF T1 | 0.27 | 0.11 | *0.015* | 0.05, 0.49 |
| Severity of overall psychopathology (CGI-S) | χ^2^(11) = 66.23, p < 0.001 | 181 | Age | -0.04 | 0.07 | 0.616 | -0.18, 0.11 |
|  |  |  | Sex | -0.63 | 0.49 | 0.199 | -1.59, 0.33 |
|  |  |  | Dataset (Bern) | -0.01 | 0.25 | 0.975 | -0.50, 0.49 |
|  |  |  | Therapy (yes) | 0.21 | 0.22 | 0.324 | -0.21, 0.63 |
|  |  |  | CGI-S T0 | 0.25 | 0.13 | 0.052 | -0.00, 0.51 |
|  |  |  | CGI-S T1 | 0.34 | 0.10 | *0.001* | 0.14, 0.53 |
| Number of suicide attempts (last 3 months) | χ^2^(11) = 18.57, p = 0.069 | 188 | Age | -0.11 | 0.20 | 0.587 | -0.49, 0.28 |
|  |  |  | Sex | -15.27 | 2839.90 | 0.996 | -5581.38, 5550.83 |
|  |  |  | Dataset (Bern) | 1.29 | 1.10 | 0.243 | -0.87, 3.46 |
|  |  |  | Therapy (yes) | 1.68 | 1.11 | 0.130 | -0.49, 3.86 |
|  |  |  | Suicide Attempts T0 | -0.10 | 1.01 | 0.324 | -2.97, 0.98 |
|  |  |  | Suicide Attempts T1 | 0.67 | 0.60 | 0.263 | -0.50, 1.84 |
| Number of days with NSSI (past month) | χ^2^(11) = 33.64, p < 0.001 | 188 | Age | -0.06 | 0.12 | 0.610 | -0.30, 0.18 |
|  |  |  | Sex | -1.74 | 1.11 | 0.116 | -3.92, 0.43 |
|  |  |  | Dataset (Bern) | 0.34 | 0.45 | 0.444 | -0.54, 1.23 |
|  |  |  | Therapy (yes) | 0.76 | 0.47 | 0.108 | -0.17, 1.68 |
|  |  |  | NSSI T0 | 0.02 | 0.03 | 0.528 | -0.04, 0.07 |
|  |  |  | NSSI T1 | 0.14 | 0.05 | *0.007* | 0.04, 0.23 |
| ***Note.*** BPD = borderline personality disorder; LoF = Level of Functioning; CGI-S = Clinical Global Impression Scale – Severity; NSSI = non-suicidal self-injury. | | | | | | | |

**References**

1. Calvo N, García-González S, Perez-Galbarro C, et al. Psychotherapeutic interventions specifically developed for NSSI in adolescence: A systematic review. *European Neuropsychopharmacology*. 2022;58:86-98. doi:10.1016/j.euroneuro.2022.02.009

2. Kaess M, Edinger A, Fischer-Waldschmidt G, Parzer P, Brunner R, Resch F. Effectiveness of a brief psychotherapeutic intervention compared with treatment as usual for adolescent nonsuicidal self-injury: a single-centre, randomised controlled trial. *Eur Child Adolesc Psychiatry*. 2020;29(6):881-891. doi:10.1007/s00787-019-01399-1

3. Buerger A, Fischer-Waldschmidt G, Hammerle F, von Auer AK, Parzer P, Kaess M. Differential Change of Borderline Personality Disorder Traits During Dialectical Behavior Therapy for Adolescents. *J Pers Disord*. 2019;33(1):119-134. doi:10.1521/pedi_2018_32_334

4. Chanen AM, Nicol K, Betts JK, Thompson KN. Diagnosis and Treatment of Borderline Personality Disorder in Young People. *Curr Psychiatry Rep*. 2020;22(5):25. doi:10.1007/s11920-020-01144-5

5. Kothgassner OD, Goreis A, Robinson K, Huscsava MM, Schmahl C, Plener PL. Efficacy of dialectical behavior therapy for adolescent self-harm and suicidal ideation: a systematic review and meta-analysis. *Psychol Med*. 2021;51(7):1057-1067. doi:10.1017/S0033291721001355

6. Wong J, Bahji A, Khalid-Khan S. Psychotherapies for Adolescents with Subclinical and Borderline Personality Disorder: A Systematic Review and Meta-Analysis. *Can J Psychiatry.* 2020;65(1):5-15. doi:10.1177/0706743719878975
